# Epidemiology and time trends of isolated greater tuberosity fractures from 1944 to 2020 – A cohort study in Malmö, Sweden

**DOI:** 10.1177/17585732251344547

**Published:** 2025-05-30

**Authors:** Anton Cederwall, Björn E Rosengren, Henrik G Ahlborg

**Affiliations:** 1 Department of Orthopedics, Lund University, Skåne University Hospital Malmo, Sweden

**Keywords:** isolated greater tuberosity fracture, proximal humeral fracture, time trend, epidemiology, incidence, Mutch classification, greater tuberosity ratio

## Abstract

**Background:**

Although up to 25% of proximal humeral fractures are isolated greater tuberosity fractures (IGTFs), comprehensive epidemiological data are lacking.

**Objectives:**

Describe the epidemiology and time trends of IGTF in Malmö, Sweden, 1944–2020.

**Methods:**

We identified IGTF in Malmö residents (≥18 years) by reviewing relevant radiology examinations during 17 sample years from 1944 to 2020. Fractures were classified according to the Mutch classification.

**Results:**

In total, 614 IGTF (60% women) were identified (mean age women 60 (SD 16), men 48 (SD 16)). Among individuals <50 years, the incidence was lower in women than men (9 95% confidence interval (CI) 7–10] vs 15 [95% CI 13–18] per 100,000 persons years) whereas the reverse was found in individuals ≥50 years (36 [95% CI 30–42] vs 19 [16–23]). No statistically significant time trends in the incidence rate were observed from year 1944 to 2020. We identified 45% avulsion-, 44% split- and 11% depression-fractures.

**Conclusions:**

IGTF is more common in men than women in age-group <50 years, while the opposite is found in age-group ≥50 years. No statistically significant time trend was observed in IGTF incidence from 1944 to 2020 in Malmö, Sweden.

## Background

Proximal humeral fractures (PHFs) have been reported as the fourth most prevalent type of non-spinal fracture among adults.^1^ Among PHF sub-groups, isolated greater tuberosity (GT) fractures (IGTFs) represent a noteworthy subset, accounting for approximately 16%–25% of all PHF cases.^2–4^ Previous studies have reported a mean age of 51–58 years in individuals with IGTF, while the proportion of women affected ranges from 42% to 60%.^5,6^

To the best of our knowledge, time trends in the incidence rate of IGTF have not been described in the current literature.

**Figure 1.**
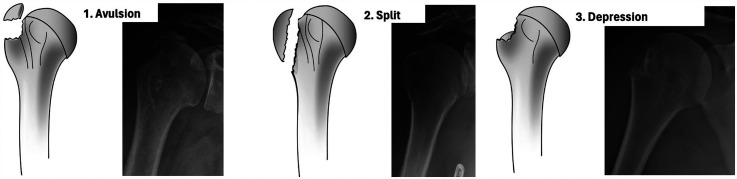
The three sub-groups of isolated greater tuberosity fractures in the Mutch classification. Published with permission from the original author.^7^

In 2014, Mutch et al. presented a classification of IGTF based on morphology, dividing the fractures into three sub-groups (Figure 1).

1. Avulsion (39%). Small fragment with horizontal fracture line.
2. Split (41%). Larger fragment with vertical fracture line.
3. Depression (20%). Inferiorly displaced.^6^

The intra- and inter-observer agreement of the Mutch classification has previously been reported as substantial, with Cohens kappa coefficient (κ) values of 0.78^6^ and 0.72^7^ for intra-observer agreement and ranging from 0.73 to 0.77^6^ and 0.63^7^ for inter-observer agreement.

**Figure 2.**
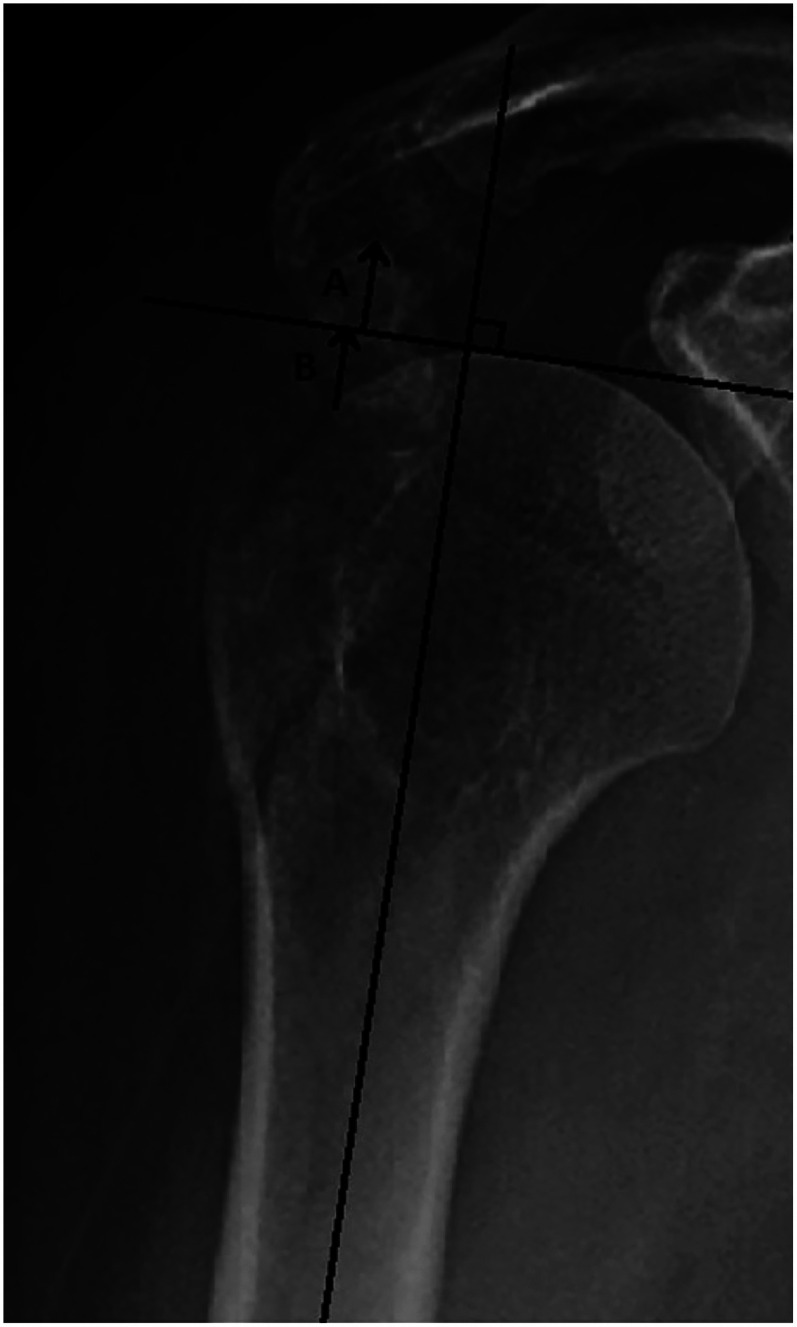
GT-ratio meassurements. The GT-ratio is used for superiorly displaced isolated GT fractures. GT-ratio = (A + B)/B. GT-ratio interpretation: < 0: Non-surgical. 0–0.5: Additional radiographs, i.e., CT. > 0.5 Surgical. Published with permission from the original author.^7^ GT: greater tuberosity; CT: computed tomography.

Mutch et al. also presented a new measurement method in 2014, the GT-ratio, which describes the superior displacement of IGTF. The ratio is calculated using the following formula: (A + B)/B. A corresponds to the distance between a tangent drawn along the most superior aspect of the humeral head and perpendicular to the humeral diaphysis and the most superior aspect of the GT fragment. B corresponds to the distance between the same tangent and the most lateral aspect of the humeral head articular surface. Mutch et al. proposed the following clinical use of the GT-ratio: <0: Non-surgical; 0–0.5: Additional radiographs, i.e., computed tomography (CT) >0.5 surgical (Figure 2). An advantage with a ratio, instead of a direct length measurement, is that it eliminates the magnification effect that may occur when measuring a distance on plain radiographs.^7^

Our primary objective was to provide a comprehensive description of IGTF patient characteristics and assess how the incidence varied according to age. The secondary objectives were to examine time trends in the sex- and age-adjusted incidence rates from 1944 to 2020 and to describe the distribution and characteristics of IGTF by the Mutch classification including the GT-ratio.

## Methods

This is an extension of a previous study where we reported time trends in fracture incidence of PHF.^8^ In the present study, we included only initial radiographs of acute IGTF from 1 January to 31 December during 17 sample years in Malmö residents ≥18 years old. We defined an IGTF as a fracture within the proximal humerus region only involving the GT. The proximal humerus region was defined as the area created by a square with sides equal to the width of the humeral head.^9^ We excluded cases with other PHF than IGTF, as well as Hill–Sachs lesions.

Malmö is a city in southern Sweden with 131,718 adult inhabitants in 1944 and 273,455 in 2020. There is only one hospital, the Skåne University Hospital, where acute fractures are treated.

From year 1944 until the turn of the millennium, both in- and out-patient radiographic examinations performed at the University Hospital in Malmö were archived in an analogue archive and subsequently in a digital archive. We reviewed all available radiographs, including anterior-to-posterior (AP) view (with the arm in internal and/or neutral rotation), lateral Y view, and axial view, for the earliest and latest available years in the analogue and digital archives, respectively. During the intervening time periods, additional years were selected to ensure an even distribution. Radiographs from 1996 to 2000 were unavailable due to flood damage. Demographic variables, including age, gender, and side of the fracture were recorded for all cases.

The first author (A.C) reviewed all radiographs coded S.F and brach.F (shoulder- and brachialis fractures) from the analogue archive for 1944–1946, 1952, 1957, 1962, 1967, 1972, 1977, 1981, 1987, 1994 and 1995. Aside from IGTF these codes included fractures in adjacent regions, for example other PHF, scapula-, clavicle- and diaphyseal humeral fractures. For 2005, 2010, 2015 and 2020, we searched the in- and outpatient hospital records for the ICD diagnose S42.20, i.e., fracture of the upper end of the humerus. The first author (A.C) reviewed the digital radiographs and recorded all IGTF for these years using Sectra IDS7, version 24.1 (Sectra AB, Teknikringen 20 SE-58330 Linköping, Sweden). Only plain radiographs were reviewed.

We chose to classify the fractures according to the Mutch classification, as it is specifically designed for IGTF, unlike the Neer and AO classifications. The Mutch classification categorizes IGTFs into avulsion, split or depression fractures (Figure 1). A split fracture was defined to extend to or distal to a line perpendicular to the humeral shaft that intersects where the articular surface curves towards the shaft while an avulsion fracture ends cranial to this line.^6^ The following criteria were used to distinguish a HSL from a depression fracture; A depression fracture is seen on an AP view with the arm in neutral rotation i.e., if the fracture only can be seen on an AP view with the arm in internal rotation it is regarded as a HSL. The detailed definitions of avulsion/split- and the HSL/depression-fractures above are based on personal communication with Dr Mutch.^10^ To ensure that the absence of AP views with the arm in neutral position did not lead to underestimation of depression fractures, we re-reviewed 100 randomly selected IGTF radiographs. The AP view with the arm in neutral rotation was present in 96% of cases.

We also noted the fracture displacement direction and distance. In cases with a combined displacement direction, for example, postero-superior, the direction with the largest displacement was used. In cases with a concomitant shoulder dislocation, the fracture displacement direction and distance were measured after reduction.

We chose to analyze age groups <50 and ≥50 years separately, based on the tradition in the field of fracture epidemiology and on previously reported epidemiological differences in PHF and IGTF between these age groups in the literature.^8,11^ Age-standardized incidence rates were calculated using direct standardization with the mean mid-year population during the 17 sample years as standard population. We used joinpoint regression analysis (Joinpoint Regression Programme, Version 4.9.1.0 – April 2022; National Cancer Institute) for analysis of time trends, presented as annual percent changes (APCs) with 95% confidence intervals (CIs).

To examine the classification reliability for the Mutch classification system, the first author re-classified 40 IGTF after one year. The fractures selected for re-classification were the 40 most recent IGTF in the digital dataset. Both the percental agreement and κ-values^12^ were calculated. 23 (57.5%) of the 40 fractures selected for reliability estimates were classified as avulsion-, 16 (40.0%) as split- and 1 (2.5%) as depression-fractures. We found a substantial intra-observer agreement, 85% and κ=0.71.

Some data were missing. 6 radiographs lacked side-marks, and 10 cases had no post-reduction radiographs.

We used Statistical Package for the Social Sciences (SPSS) v28.0 (IBM SPSS Statistics for Macintosh, Version 28.0. Armonk, NY: IBM Corp) and Microsoft Excel v16.67 (Microsoft Corp., Redmond, WA, USA) for database management and statistical analysis.

Ethical approval of the study was obtained prior to study start from the regional ethical review board of Lund University (LU 2012-394).

## Results

During 17 sample years that included 3231,161 person years, we found 614 IGTF in individuals ≥18 years, 366 (60%) in women and 248 (40%) in men. This corresponds to an overall incidence rate of 19 per 10^5^ person years (22 per 10^5^ person years in women and 16 per 10^5^ persons years in men). Mean age was 55 years (SD 17, range 18–97): women 60 years (SD 16, range 19–97) and men 48 years (SD 16, range 18–89). Patient and fracture characteristics are presented in Table 1.

**Table 1.** Patient and fracture characteristics, including count, mean age, fractured side, dislocation and displacement distance stratified by sex of isolated greater tuberosity fractures in adults during 17 sample years 1944–2020 in Malmö, Sweden.

		Women	Men	Total
Count *n* (row%)		366 (60%)	248 (40%)	614 (100%)
Mean age years (SD)		60 (16)	48 (16)	55 (17)
Fractured side *n* (column%)				
*n* = 608	Right	173 (48%)	132 (54%)	305 (50%)
	Left	189 (52%)	114 (46%)	303 (50%)
Glenohumeral dislocation *n* (column%)				
*n* = 614	Yes	67 (18%)	51 (21%)	118 (19%)
	No	299 (82%)	197 (79%)	496 (81%)
Displacement group *n* (column%)				
*n* = 604	<5 mm	284 (79%)	181 (74%)	465 (77%)
	≥5 mm	76 (21%)	63 (26%)	139 (23%)

### Sex-specific incidence in younger and older adults

**Figure 3.**
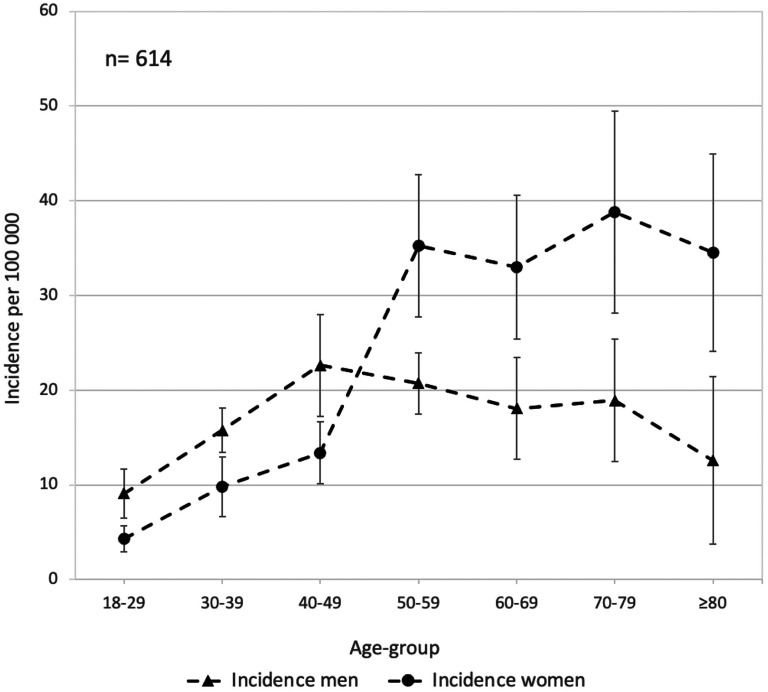
Sex-specific sample year average incidence per 100,000 adults of isolated greater tuberosity fractures, categorized by age groups, in Malmö, Sweden 1944–2020. Bars represent 95% confidence intervals.

Details on sex and age-group specific IGTF incidence are presented in Figure 3. In the age group <50 years we observed a lower incidence in woman than men (9 per 10^5^ person years [95% CI 7–10] vs 15 per 10^5^ persons years [95% CI 13–18]). Conversely, in age-group those aged ≥50 years we observed a higher incidence in women than men (36 per 10^5^ person years [95% CI 30–42] vs 19 per 10^5^ persons years [95% CI 16–23]). The overall, men and women combined, age-group-specific IGTF incidence is presented in Supplemental Figure 1.

In women, the incidence was lower in age-group those aged <50 years compared to age-group those aged ≥50 years (9 per 10^5^ persons years [95% CI 7–10] vs 36 per 10^5^ person years [95% CI 30–42]). In contrast, incidence was similar in men aged <50 years and ≥50 years (15 per 10^5^ persons years [95% CI 13–18] vs 19 per 10^5^ person years [95% CI 16–23]).

### Time trends in age- and sex-adjusted incidence

**Figure 4.**
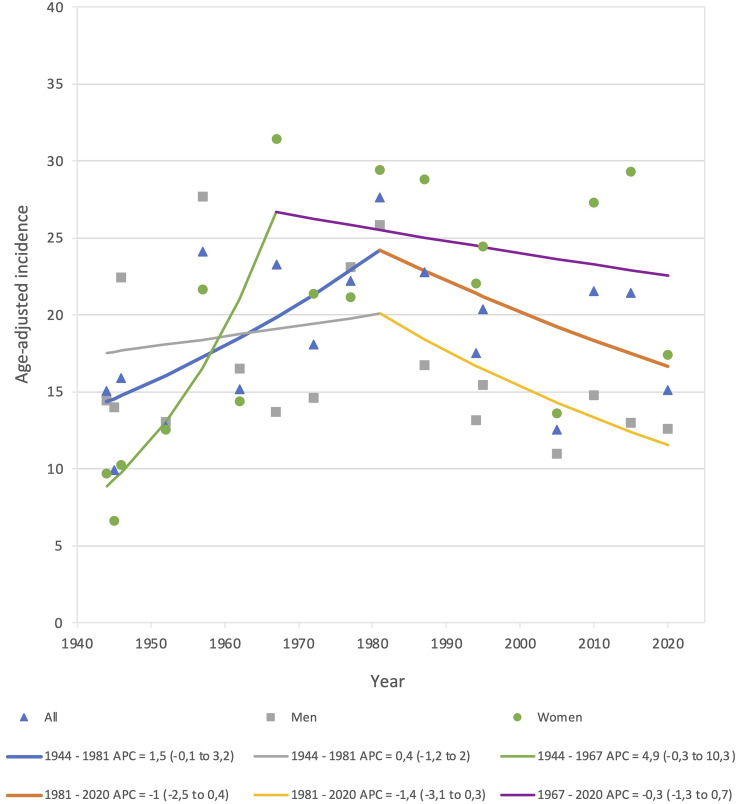
Overall (sex- and age-adjusted) and sex-specific (age-adjusted) incidence rate time trends of isolated greater tuberosity fractures in Malmö, Sweden in adults during 17 sample years 1944–2020 presented as APC with 95% confidence interval (95% CI). Lines represent joinpoint time-trends, details regarding these are presented below the chart. APC: annual percent change; CI: confidence interval.

In Figure 4, we visually observed an overall peak in incidence during the 1980s, with a separate peak for women during the 1960s, and for men in the 1980s. However, we did not identify any statistically significant time trends in the overall (sex- and age-adjusted) or sex-specific (age-adjusted) IGTF incidence between 1944 and 2020. The overall and sex-specific count of IGTF, as well as the population at risk, per sample year are presented in Supplemental Table 1.

### Fracture characteristics including classification

In total, 19% of all IGTF was accompanied by a concomitant shoulder dislocation, 46% of all IGTF were displaced superiorly and 23% were displaced ≥5 mm (Table 2).

**Table 2.** Mutch-type-specific patient and fracture characteristics regarding sex, age, displacement direction, dislocation, displacement extent group and GT-ratio-group in adults during 17 sample years 1944–2020 in Malmö, Sweden.

		Avulsion	Split	Depression	Total
Sex *n* (%)					
*n* = 614	Female (column%)	154 (55%)	181 (68%)	31 (46%)	366 (60%)
	Male (column%)	124 (45%)	87 (32%)	37 (54%)	248 (40%)
	Total (row%)	278 (45%)	268 (44%)	68 (11%)	614 (100%)
Mean age year (SD)					
*n* = 614	Women	58 (17)	60 (15)	68 (13)	60 (16)
	Men	46 (15)	50 (16)	47 (18)	48 (16)
	Total	53 (17)	56 (16)	56 (19)	55 (17)
Displacement direction *n* (column%)					
*n* = 604	Superior	151 (55%)	126 (48%)	0 (0.0%)	277 (46%)
	Posterior	24 (9%)	34 (13%)	0 (0.0%)	58 (10%)
	Inferior	10 (4%)	24 (9%)	66 (97%)	100 (17%)
	None	88 (32%)	75 (28%)	2 (3%)	165 (27%)
	Lateral	0 (0%)	4 (2%)	0 (0%)	4 (1%)
Glenohumeral dislocation *n* (column%)					
*n* = 614	Yes	53 (19%)	43 (16.0%)	22 (32%)	118 (19%)
	No	225 (81%)	225 (84.0%)	46 (68%)	496 (81%)
Displacement extent group *n* (column%)					
*n* = 604	<5 mm	207 (76%)	196 (75%)	62 (91%)	465 (77%)
	≥5 mm	66 (24%)	67 (25%)	6 (9%)	139 (23%)
GT-ratio^a^-group *n* (column%)					
*n* = 276	0–0.5	104 (69%)	99 (78%)	n/a	202 (73%)
	>0.5	46 (31%)	28 (22%)	n/a	74 (27%)

IGTF: isolated greater tuberosity fracture; GT: greater tuberosity; CT: computed tomography.

^a^ GT-ratio: measuring superior displacement of IGTF; 0–0.5: CT recommended; >0.5: surgery.

According to the Mutch classification we identified 45% avulsion-, 44% split- and 11% depression-fractures (Table 2). We observed 55% women in the avulsion fracture group and 68% women in the split fracture group (Table 2). Individuals with avulsion and split fractures exhibited similar mean ages and fracture characteristics (displacement direction, percent dislocated, displacement extend group and GT-ratio). In contrast, for individuals with depression fractures we saw different fracture characteristics (higher proportion of individuals in the dislocated and the less displaced group) and a male dominance (54%), setting them apart from avulsion- and split-fractures (Table 2).

## Discussion

We found 614 IGTF in adults during 17 sample years from 1944 to 2020 in Malmö, Sweden. IGTF were more common in men than women in age-group <50 years but conversely more common in women than men in age-group ≥50 years. For women, but not men, the incidence of IGTF was higher in ages ≥50 years compared to <50 years. We found no statistically significant time trend in the sex- and age-adjusted incidence rate of IGTF between 1944and 2020. We identified 45% avulsion-, 44% split- and 11% depression-fractures.

In 2005, Kim et al. reported the age-distribution of 115 IGTF. Their result indicated that the incidence of IGTF increased age until age-group 40–49, after which it declined. No sex-specific data were reported.^11^ We found a similar pattern in men but in women the incidence was lower in age-group <50 years than in age-group ≥50 years.

Osteoporosis is a well-known risk factor for PHF.^13^ To our knowledge, the relationship between osteoporosis and the risk of an IGTF is not as well established. Our finding, indicating a higher incidence of IGTF among women with advancing age, particularly evident between the age-groups 40 to 49 and 50 to 59 (Figure 3), may however imply an association between IGTF and osteoporosis. The age-specific incidence pattern in women with a four-fold increase after age 50 years is similar to what earlier have been shown for distal radius fracture.^14^ Future research is needed to further explore this relationship.

Kim et al. also found that individuals with an IGTF tend to be younger and to a predominantly male compared to individuals with other PHF. They reported demographic differences between individuals with IGTF (*n* = 115) and those with other PHF (*n* = 495) from South Korea 1989–2004. Individuals with an IGTF had a mean age of 42.8 years and 32.2% were women. The same numbers for individuals with a non-IGTF PHF were 54.2 years and 68.1% women.^11^

Based on data from a previous study^8^ by our research group, persons with a non-IGTF PHF were predominantly women (77%) with an overall mean age of 70 years (SD 14). In the current study of IGTF, 60% were women with an overall mean age of 55 years (SD 17). This aligns with the results reported by Kim et al.,^11^ despite the lower mean age in the Korean study, and indicates that individuals with an IGTF tend to be younger and, to a greater extent, male compared to individuals with other PHF.

We were unable to find any statistically significant time trends in the age-adjusted incidence of IGTF 1944–2020. We, however, saw a tendency towards an increase in the age- and sex-adjusted incidence, men and women combined, between 1944 and 1981, APC = 1.5 (−0.1 to 3.2) and thereafter some indications of a decrease until 2020 APC = −1 (−2.5 to 0.4) (Figure 4). To the best of our knowledge, no other long-term time trend study of IGTF exists.

The distribution of fractures according to the Mutch classification system in our study (45% avulsion, 44% split and 11% depression) is comparable to figures from Mutch et al. (39% avulsion, 41% split and 20% depression)^6^ and Razaeian et al.^15^ (56% avulsion, 34% split and 10% depression).

We found 23% IGTF displaced ≥5 mm. Mutch et al. reported 31% displaced ≥5 mm^6^ and a systematic review published in 2017 reported 41% displaced >5 mm.^5^ In the present study, the fracture displacement distance was measured after reduction in the situation of a concomitant shoulder dislocation. When the measurements were done is not stated in the systemic review^5^ or in the Mutch et al.^6^ study, making this a possible contributor to the differences.

We found a concomitant shoulder dislocation in 19% of all IGTF and in 32% of depression fractures. Mutch et al.^6^ reported a shoulder dislocation in 28% of all IGTF and in 46% of depression fractures. We defined a shoulder dislocation as the presence of radiographs displaying a dislocated humeral head. It is however, reasonable to assume that some dislocations in our study were reduced before the radiology examination, resulting in a possible underestimation of dislocations in our study.

We found a substantial intra-observer agreement in our reliability analysis, κ=0.71, similar to Mutch et al.^6^ in 2014 (κ=0.78)and Razaeian et al.^15^ in 2021 (κ=0.72). The fractures selected for reliability analysis displayed a similar Mutch classification distribution as the total dataset, making it reasonable to assume that these 40 fractures can be considered representative for the total dataset.

Strengths of the present study include that all fractures were reviewed and classified by a single orthopaedic consultant with subspeciality in shoulder surgery and the substantial intra-observer agreement. The study period spanning almost 80 years could also be considered a strength and facilitates detection of major time trends during a relatively long period.

It is important to acknowledge that the findings in the current study may not be readily applicable or generalizable to vastly different settings, such as developing countries. Sweden has been reported to have one of the highest fracture incidences globally.^16^ Therefore, while our results may not be universally applicable, they offer valuable insights into fracture epidemiology and detection of emerging trends.

Our decision to measure only initial fracture displacement may be considered a weakness, as further displacement may occur. According to a study by Bockmann et al. in 2019^17^ 23% (*n* = 19) of initially minimally displaced (less than 5 mm) IGTF were displaced more than 5 mm at a mean follow-up at 8 days.

The displacement direction is often a combination of directions.^18^ However, we only registered the direction with the largest displacement. This simplification is similar to a previous study reporting on the displacement direction of IGTF,^19^ but could none the less be considered a weakness.

Another weakness of the present study, as with other studies on IGTF,^6,11^ is the use of plain radiographs for classification and measurements. Posterior displacement may then be difficult to detect and very challenging to measure. Studies based on CT-scans would probably facilitate more accurate detection and measurement of posterior displacement.

## Conclusion

In this, to the best of our knowledge, largest study on the epidemiology of IGTF we found that IGTF is more common in men than women in age-group <50 years while the opposite is true ≥50 years. We found no statistically significant time trend in the sex- and age-adjusted incidence rate of IGTF between 1944 and 2020. We found 45% avulsion, 44% split and 11% depression fractures according to the Mutch classification.

## Supplemental Material

sj-tiff-1-sel-10.1177_17585732251344547 - Supplemental material for Epidemiology and time trends of isolated greater tuberosity fractures from 1944 to 2020 – A cohort study in Malmö, Sweden Supplemental material, sj-tiff-1-sel-10.1177_17585732251344547 for Epidemiology and time trends of isolated greater tuberosity fractures from 1944 to 2020 – A cohort study in Malmö, Sweden by Anton Cederwall, Björn E Rosengren and Henrik G Ahlborg in Shoulder & Elbow

sj-tiff-2-sel-10.1177_17585732251344547 - Supplemental material for Epidemiology and time trends of isolated greater tuberosity fractures from 1944 to 2020 – A cohort study in Malmö, Sweden Supplemental material, sj-tiff-2-sel-10.1177_17585732251344547 for Epidemiology and time trends of isolated greater tuberosity fractures from 1944 to 2020 – A cohort study in Malmö, Sweden by Anton Cederwall, Björn E Rosengren and Henrik G Ahlborg in Shoulder & Elbow
