# Supplementary figures and images for: Epidemiology and time trends of isolated greater tuberosity fractures from 1944 to 2020 – A cohort study in Malmö, Sweden
Source: Shoulder Elbow. 2025 May 30;18(2):326–33. doi: 10.1177/17585732251344547 (PMC12126461; doi:10.1177/17585732251344547)

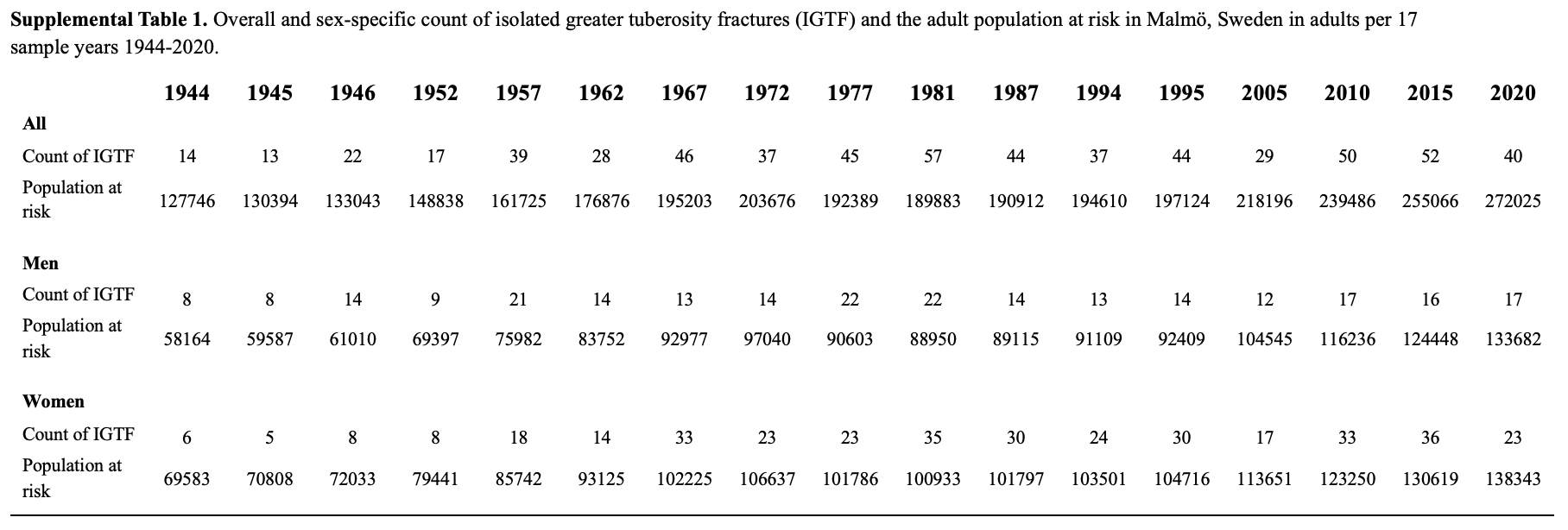

Supplement: sj-tiff-1-sel-10.1177_17585732251344547 - Supplemental material for Epidemiology and time trends of isolated greater tuberosity fractures from 1944 to 2020 – A cohort study in Malmö, Sweden [file sj-tiff-1-sel-10.1177_17585732251344547.tiff]

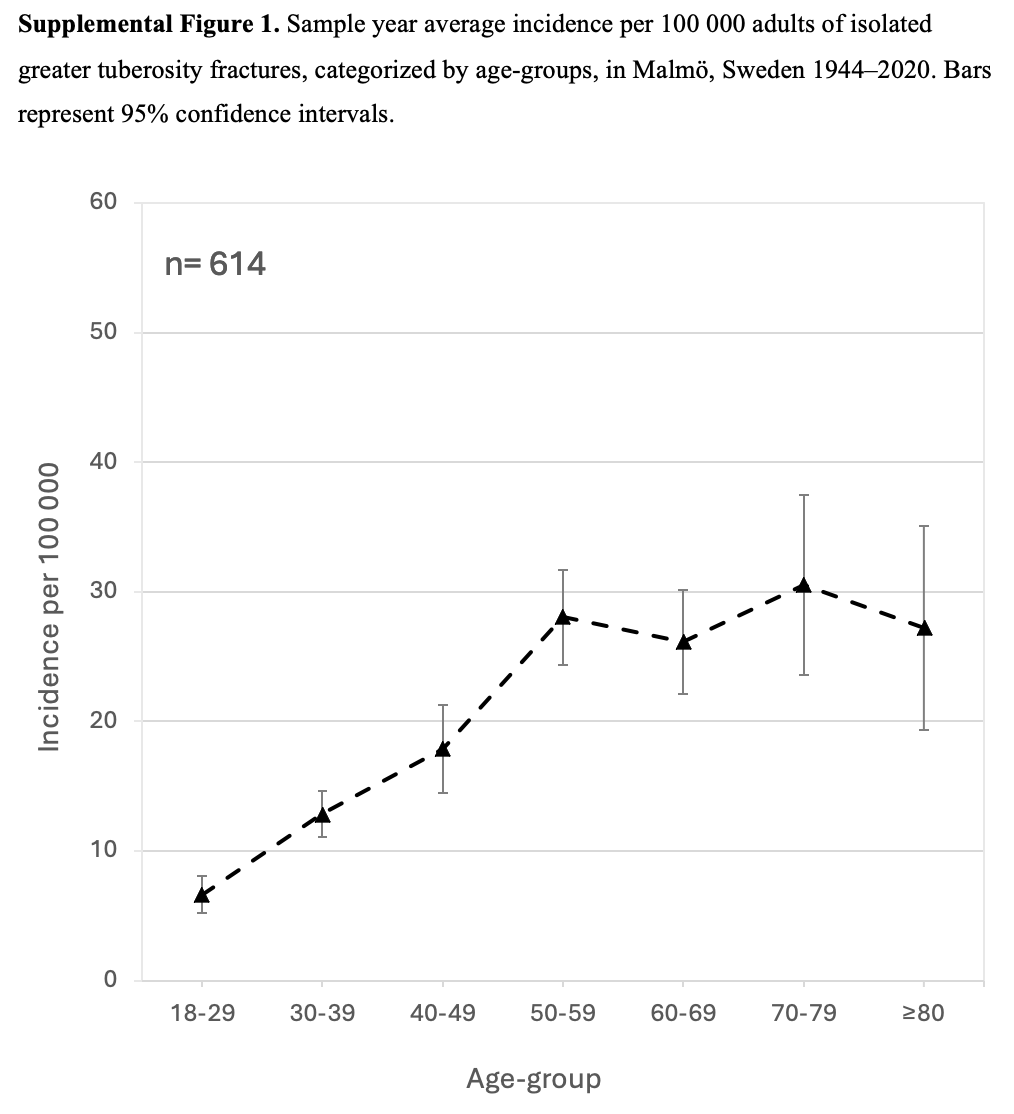

Supplement: sj-tiff-2-sel-10.1177_17585732251344547 - Supplemental material for Epidemiology and time trends of isolated greater tuberosity fractures from 1944 to 2020 – A cohort study in Malmö, Sweden [file sj-tiff-2-sel-10.1177_17585732251344547.tiff]
